# Reduced Expression of Immune Mediators by T-Cell Subpopulations of Combat-Exposed Veterans With Post-Traumatic Stress Disorder

**DOI:** 10.3389/fpsyt.2019.00693

**Published:** 2019-09-18

**Authors:** Ying Xiong, Zhewu Wang, M. Rita I. Young

**Affiliations:** ^1^Department of Otolaryngology–Head and Neck Surgery, Medical University of South Carolina, Charleston, SC, United States; ^2^Mental Health Service, Ralph H. Johnson VA Medical Center, Charleston, SC, United States; ^3^Department of Psychiatry, Medical University of South Carolina, Charleston, SC, United States; ^4^Research Service, Ralph H. Johnson Veterans Affairs Medical Center, Charleston, SC, United States

**Keywords:** granzyme B, immune, interferon-γ, post-traumatic stress disorder, T-cell, veterans

## Abstract

Post-traumatic stress disorder (PTSD) has been suggested to be associated with an inflammatory immune state, although few studies have examined peripheral blood lymphocytes in subjects that have PTSD and compared immune parameters to subjects that experienced similar trauma, but did not develop PTSD. An exploratory approach was undertaken to compare phenotypes of blood CD4^+^ and CD8^+^ subpopulations and their expression of immune mediators between Veterans of the Iraq and Afghanistan wars who experienced similar levels of combat, with some developing PTSD and other not. The results of this study did not demonstrate evidence of enhanced immune activation of peripheral blood lymphocytes. Instead, the results showed a decline in expression of the pro-inflammatory mediator IFN-γ and the cytotoxin granzyme B in CD8^+^ subpopulations from Veterans with PTSD. While the reductions in expression of IFN-γ and granzyme B did not reach statistical significance when examining the CD8^+^ cell population as a whole, the declines were significant when examining the CD8^+^ cell subpopulations, with different mediators being reduced in different subpopulations. The most prominent decline in IFN-γ expression was by the unconventional CD8^dim^CD3^+^ T-cell subpopulation that has been associated with chronic infection and immune fatigue. The decline in granzyme B was most prominent in the NK-containing CD8^dim^CD3^-^ subpopulation of Tcells. Consequently, analysis of blood leukocyte subpopulations, rather than bulk lymphocyte groups, reveals a dampened level of immune reactivity in combat-exposed Veterans with PTSD compared to combat-exposed Veterans without PTSD.

## Introduction

Post-traumatic stress disorder (PTSD) is a debilitating condition that afflicts both military personnel and civilians. According to the Department of Veterans Affairs, approximately 15% of military returning from conflicts in Iraq and Afghanistan have PTSD (1). PTSD is not limited to combat military, but occurs in civilians following traumatic events such as earthquakes, flooding or sexual assault (2–4). Characteristics of PTSD include re-experiencing the traumatic events, avoidance of traumatic event reminders, hyper-arousal and negative feelings and thoughts.

There have been suggestions that PTSD is associated with a pro-inflammatory immune state, although results of such studies have not been uniform. For example, a large meta-analysis of blood cytokine levels in subjects with PTSD versus healthy controls showed significantly increased levels of IL-1β, IL-6, TNF-α, and IFN-γ in subjects with PTSD (5). Veterans with late-life onset of PTSD had increased serum levels of the inflammatory marker C-reactive protein (CRP) (6). Plasma levels of the inflammatory mediator TNF-α and the chemokines CXCL-10 and CCL13 were higher in the plasma of subjects with civilian PTSD compared to levels in healthy controls (7). Patients that had been exposed to a deadly earthquake had higher blood levels of pro-inflammatory cytokines IL-1 and TNF-α than subjects that were similarly exposed but did not have PTSD (8). Several immune mediators, including the pro-inflammatory mediators G-CSF and CCL5 and the anti-inflammatory mediator IL-4 were increased in plasma of individuals with long-term PTSD following childhood exposure to suicide bomb terror (9). A study from our laboratory comparing plasma cytokine levels in combat Veterans with PTSD versus combat Veterans without PTSD, showed increased levels of the pro-inflammatory mediators IL-2, IL-6 and IL-17, but decreased levels of the anti-inflammatory mediator IL-4 in those with PTSD (10). In an assessment of male Croatian combat Veterans, no difference was demonstrated in serum levels of IL-1β, IL-2, TNF-α, IFN-γ, IL-4, or IL-6, but a slight decrease was demonstrated in the chemoattractant cytokine IL-8 in Veterans with PTSD (11). A study of inpatients in a psychiatric center showed serum levels of IL-1β, CCL2, and TNF-α were not increased in subjects with PTSD compared to subjects with other mental health disorders (12). In a study in which levels of IL-6 in plasma were measured over a 24-h time period, no differences were seen between combat patients with PTSD, combat controls and healthy controls, but both groups that experienced combat had a blunted IL-6 circadian rhythm (13).

Although studies have suggested variations in blood cytokine levels in subjects with PTSD, only a few studies have conducted phenotypic or functional analyses of immune cell subpopulations. T-cell subpopulations would include CD4^+^ T-helper or immune inhibitory T-regulatory (Treg) cells, and CD8^+^ cells with cytotoxic and effector functions. Several CD8^+^ subpopulations have been described, including conventional CD8^+^ cells, unconventional CD8^+^ cells associated with chronic infection and immune fatigue, and NK-containing populations of CD8^+^ cells (14–16). Phenotypic analysis of blood leukocytes of females with PTSD that experienced childhood sexual trauma showed no difference in the proportions of CD4^+^ T-cells, CD8^+^ T-cells or NK cells compared to levels in healthy controls. However, an increase in the ratio of memory to naïve T-cells was observed, suggesting increased lymphocyte activation (17). In a study of Detroit residents, the ratio of CD4 to CD8 T-cells was lower in subjects with PTSD compared to Detroit residents without PTSD, but the proportion of effector to naïve CD8 cells was greater in PTSD subjects, suggestive of immunological aging (18). A lower proportion of CD4^+^ T-cells was also seen in a study of Croatian male combat Veterans compared to levels in healthy controls (19). This study also showed that subjects with PTSD have a lower proportion of Treg, suggesting that a reduction in immune suppressive capability could be a mechanism for increased immune reactivity. A study of combat-exposed Veterans with and without PTSD showed an increased in atypical CD56^-^CD16^+^ NK cells, which are considered to be dysfunctional, and a decrease in functional CD56^bright^CD16^-^ NK cells in subjects with PTSD (20). Veterans with rheumatoid arthritis that also had PTSD had increased serum levels of a spectrum of cytokines compared to those without PTSD, suggesting that PTSD could heighten the inflammatory state of rheumatoid arthritis (21). A prospective study demonstrated altered pre-deployment glucocorticoid control of LPS-induced monocyte TNF-α secretion and altered pre-deployment control of PHA-stimulated T-cell proliferation in military personnel that developed PTSD during deployment, suggesting that immune sensitivity to glucocorticoids prior to deployment could be a marker for development of PTSD as a result of deployment (22).

Since studies have typically compared immune parameters of PTSD subjects to healthy controls that did not experience similar trauma, and have typically shown increased blood levels of pro-inflammatory cytokines, there remains a need for examination of the immune phenotype of subjects that experienced similar levels of trauma, with some having developed PTSD and others not. The present study conducted a phenotypic analysis of peripheral blood leukocytes of a tightly controlled population of subjects, namely Veterans of the ongoing wars in Iraq and Afghanistan who experienced similar levels of combat, with some developing PTSD and others not developing PTSD.

## Materials and Methods

### Research Subjects

Veterans with and without PTSD were recruited from the Ralph H. Johnson VA Medical Center in Charleston, SC. The study was approved by the Institutional Review Board of record. The study was explained to Veterans and they provided voluntary consent to participate in the study. Both males and females were recruited without regard to gender. Eligibility criteria to participate in the study included combat Veterans of the OEF/OIF arenas in Iraq or Afghanistan, ages 19 to 60, in good physical health. Veterans were excluded if they had lifetime or ongoing serious mental health disorders such as schizophrenia or bipolar disorder, serious traumatic brain injury, substance abuse, immune-affecting disorders such as cancer or HIV, or if they were pregnant. If Veterans did not have a clinical diagnosis of PTSD that was associated with their military service, they were administered the Clinician-Administered PTSD Scale for DSM-5 (CAPS-5) so as to triage subjects into the control or PTSD groups. The level of combat was measured by the Combat Exposure Scale (CES). Subjects enrolled in the study provided a sample of peripheral blood and, at that point, their participation in the study was complete.

### Analysis of Peripheral Blood Leukocytes

Peripheral blood samples from control and PTSD subjects were collected and peripheral blood mononuclear cells (PBMCs) were isolated by density gradient centrifugation (Histopaque^®^-1077, Sigma, St Louis, MO). PBMCs were immediately frozen and stored in a liquid nitrogen tank until further use. In preparation for flow cytometric analysis, the stored cells were thawed and stimulated at 37°C for 5 h with Cell Stimulation Cocktail plus protein transport inhibitors (Invitrogen, Carlsbad, CA), containing 50 ng/ml PMA, 1 μg/ml ionomycin, 10.6 µM Brefeldin A, and 2 µM Monensin.

Fluorescence-conjugated mouse anti-human antibodies and kits were all purchased from BD Biosciences (San Diego, CA). Anti-CD3-APC-Cy7 (SK7), anti-CD4-PerCP-Cy5.5 (RPA-T4), and anti-CD8-PE (RPA-T8) antibodies were used to determine the cell surface expressions of CD3, CD4, and CD8. Following cell fixation and permeabilization with Cytofix/Cytoperm kit, anti-IFN-γ-FITC (B27) and anti-granzyme B-Alexa Fluor 647 (GB11) antibodies were used to measure the intracellular levels of IFN-γ and granzyme B. The Human T17/Treg Phenotyping kit was used for intracellular Foxp3 staining according to the manufacturer’s instructions. The stained cells were phenotypically analyzed using a FACSCanto flow cytometer (BD Biosciences).

### Statistical Analysis

The Shapiro-Wilks test was used to determine normality of the data sets. However, the Kolmogorov-Smirnov test was instead used to determine normality of the distribution of ages of the subjects in the two groups as there were identical values in each group. Most data sets were normally distributed. For data sets that were normally distributed, the Student’s *t* test was used to analyze differences between values for control and PTSD subjects. For the few data sets that were not normally distributed, the Mann-Whitney *U* nonparametric test was used to calculate the significance of differences. Since the sample size differed between the control and PTSD groups of Veterans, the Hedge’s *g* test was used to determine the effect size measure. To determine the significance of differences in gender, race and other co-morbidities among the two groups of Veterans, the Fisher’s exact test was used. Differences were considered to be statistically significant at the 95% confidence interval. The Spearman’s rank correlation test was used to determine if there was any association between age and the immune parameters that were measured. All the data were presented as mean ± SD.

## Results

### Demographics of Research Veterans

Thirty-two combat-exposed Veterans of the Iraq and Afghanistan wars were enrolled into the study: 13 controls without PTSD and 19 with PTSD (**Table 1**). While there were more females in the PTSD group, the difference was not statistically significant. Nevertheless, the possible impact of gender differences among groups was assessed by determining if the same conclusions were reached if data were analyzed for males only. This revealed that there was not an effect of gender on immunological parameters (further described below). There were no significant differences in the distribution of races among the two groups. The level of combat exposure for these two groups of Veterans was similar based on the CES. The proportion of Veterans that had co-morbidities, including depression and immunologic disorders, were also similar. A prior history of an alcohol use disorder was not significantly different, although Veterans with ongoing alcohol or other drug use disorders were excluded from study participation. What was different was the age, with Veterans without PTSD being older than those with PTSD (*p* = 0.007). A Spearman’s rank correlation analysis showed there was no correlation between ages of the Veterans and any immunological parameters. To further determine if the age difference among the two groups impacted on immunological parameters, values for subjects that were age-matched were analyzed. This allowed analysis of 11 age-matched subjects per group (further describe below).

**Table 1:** Descriptive information on research subjects.

	Controls	PTSD	p
	(n = 13)	(n = 19)	
Age (range)	45.6 ± 9.5 (30-60)	36.5 ± 6.8 (26-50)	0.007
Gender Males Females	11 (84.6%) 2 (15.4%)	12 (63.2%) 7 (36.8%)	NS NS
Race Caucasian African American Hispanic Other	4 (30.8%) 6 (46.2%) 3 (23.1%) 0 (0.0%)	10 (52.6%) 5 (26.3%) 2 (10.5%) 2 (10.5%)	NS NS NS NS
Depression	4 (30.8%)	6 (31.6%)	NS
Prior alcohol use disorder	2 (15.4%)	2 (10.5%)	NS
Immunological disorders (total) Psoriasis Allergic rhinitis Arthritis	4 (30.8%) 2 (15.4%) 2 (15.4%) 0 (0%)	6 (31.6%) 2 (10.5%) 3 (15.8%) 1 (5.3%)	NS
Combat exposure scale	22.4 ± 7.2	23.2 ± 9.4	NS

### Comparison of Blood CD4^+^ and CD8^+^ Cell Populations Between Combat Veterans With PTSD or Without PTSD

**Figure 1:**
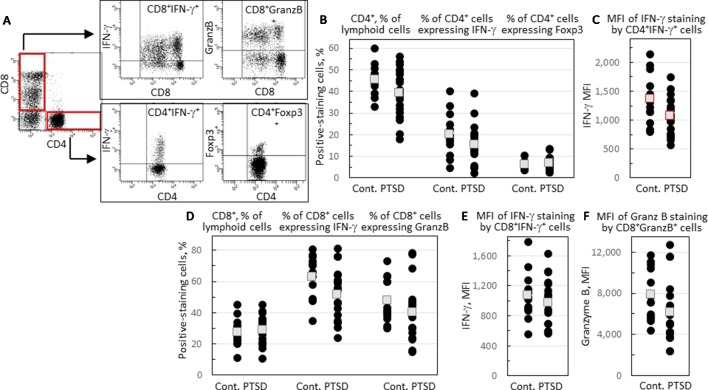
Comparison of levels of CD4^+^ and CD8^+^ cells and the proportion of these cells that express immune mediators. **(A)** A representative illustration of the gating strategy. Lymphoid cells were stained for CD4 and CD8 expression and identified in red-outlined quadrants as CD4^+^CD8^-^ or CD8^+^CD4^-^ cells. Gated CD4^+^CD8^-^ cells were further analyzed for IFN-γ and Foxp3 staining, and gated CD8^+^CD4^-^ cells analyzed for IFN-γ and granzyme B (GranzB) staining. **(B)** The percentages of CD4^+^CD8^-^ cells, and the percentages of gated CD4^+^CD8^-^ cells that express IFN-γ or Foxp3. **(C)** The mean fluorescence intensity (MFI) of IFN-γ staining by gated CD4^+^CD8IFN-γ^+^ cells. **(D)** Percentages of CD8^+^CD4^-^ cells, and the percentages of gated CD8^+^CD4^-^ cells that express IFN-γ or granzyme B. **(E)** MFI of IFN-γ staining by gated CD8^+^CD4^-^IFN-γ^+^ cells. **(F)** MFI of granzyme B staining by gated CD8^+^CD4^-^ cells. Shown as black circles are data for each research subject, with the mean values shown as squares. Mean values that are statistically different between Veterans with and without PTSD are shown as red squares.

Peripheral blood CD4^+^ cells and their expression of immune mediators were compared for combat Veterans with PTSD and Veterans exposed to similar levels of combat that did not have PTSD. The gating strategy for phenotypic analysis of CD4^+^CD8^-^ and CD8^+^CD4^-^ cells is shown in **Figure 1A**. There was not a statistically significant difference in the proportion of leukocytes that were CD4^+^ among the two groups, although there was a strong tendency that neared significance toward a reduced level in Veterans with PTSD (*p* = 0.057; **Figure 1B**). The proportion of CD4^+^ cells that co-expressed pro-inflammatory mediator IFNγ tended to be lower, but was not significantly different between the two groups (**Figure 1B**). However, the intensity of IFN-γ expression by the CD4^+^IFNγ^+^ cells was statistically significantly lower in Veterans with PTSD compared to those without (*p* = 0.049; **Figure 1C**). Sample size calculation indicated that 25 subjects would be required per group for statistical significance, but a large effect size (Hedge’s *g* = 0.79) was attained with 13 controls and 19 PTSD subjects, highlighting the significance of the difference between subjects with or without PTSD. Since there was a difference in the proportion of females among the two groups, the intensity of IFN-γ staining by CD4^+^ cells was compared for only male subjects in the PTSD and control groups. Removing values for females resulted in a loss of significance in the difference between PTSD and controls, although the intensity of IFN-γ staining by CD4^+^ cells remained lower for the PTSD group (1,048 ± 347) compared to the controls (1,320 ± 383). To determine the impact of the difference in age between the two groups, values were analyzed only for age-matched subjects. This resulted in a loss of power for the difference in intensity of IFN-γ staining by CD4^+^ cells, although the values remained lower for the PTSD group (1,054 ± 392) compared to the controls (1,302 ± 409).

The proportion of CD4^+^ cells expressing the inflammatory mediator IL-17 was low in both groups (mean < 1%, data not shown). Expression of Foxp3, a marker for the immune inhibitory Treg cells, was also measured, with no difference being observed in the proportion of CD4^+^ T-cells expressing Foxp3 between Veterans with and without PTSD (**Figure 1B**).

A comparison of blood levels of CD8^+^ T-cells showed a near significant reduction in the proportion of CD8^+^ cells expressing IFN-γ (*p* = 0.054; **Figure 1D**). The intensity of IFN-γ expression by CD8^+^IFN-γ^+^ cells was similar for Veterans with or without PTSD (**Figure 1E**). Expression of the cytotoxin granzyme B by CD8^+^ cells was also measured. There was not a significant difference in the proportion of CD8^+^ cells expressing granzyme B (**Figure 1D**), but there was a near significant reduction in the intensity of granzyme B expression by CD8^+^granzyme B^+^ cells (*p* = 0.066; **Figure 1F**) of Veterans with PTSD compared to those without PTSD.

### Comparison of CD8+ Subpopulations Between Combat-Exposed Veterans With PTSD and Those Without PTSD

**Figure 2:**
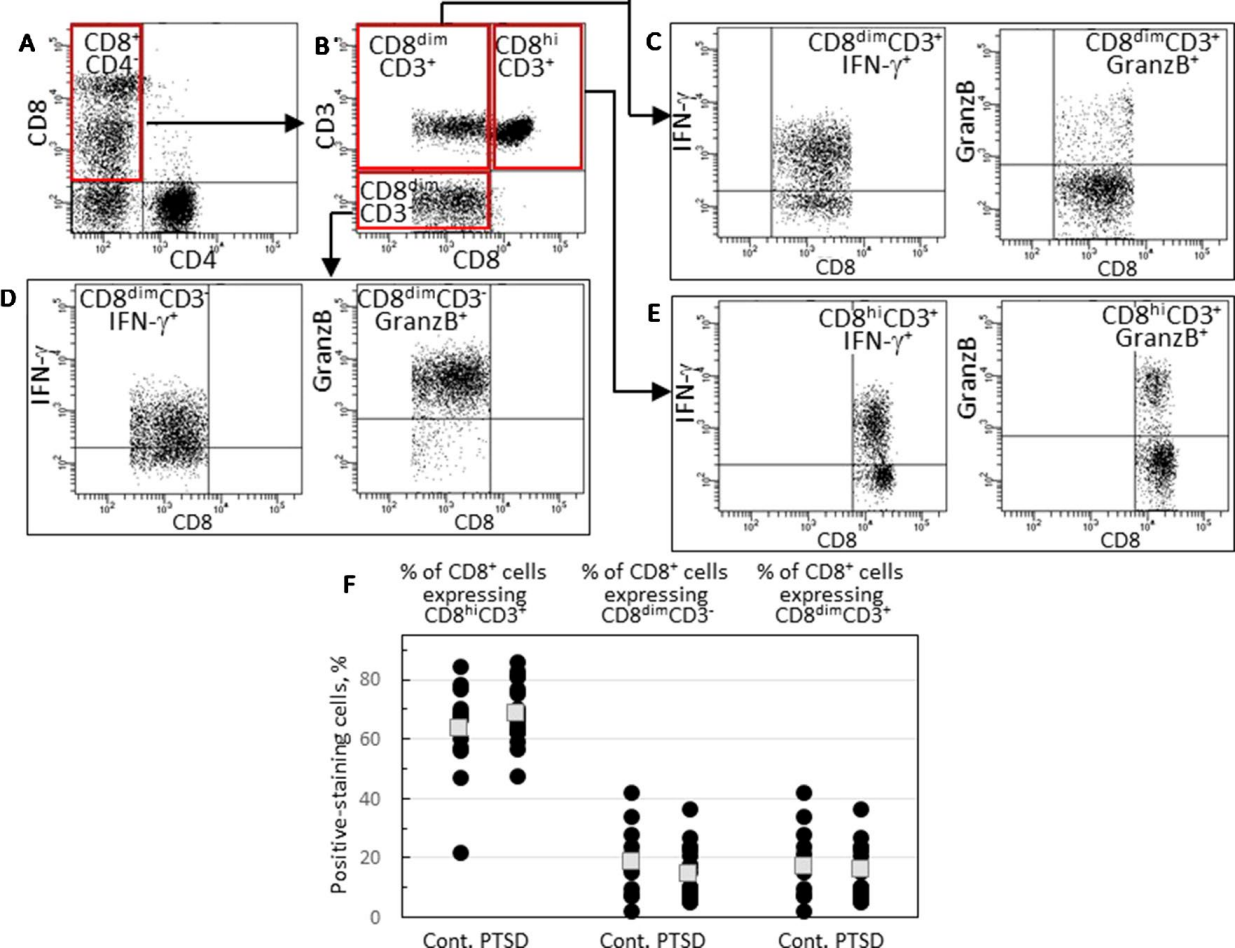
Identification and analysis of three CD8^+^ cell subpopulations. **(A)** A typical scattergram of single lymphoid cells stained for CD8 or CD4. Cells staining positive for CD8 and negative for CD4 are identified in the quadrant that is outlined in red. **(B)** Gated CD8^+^CD4^-^ cells were analyzed for CD3 and CD8 staining and were identified by the red-outlined quadrants as cells that stain bright for CD8 and stain for CD3 (CD8^hi^CD3^+^), cells that stain dimly for CD8 and stain for CD3 (CD8^dim^CD3^+^) and cells that stain dimly for CD8 but do not stain for CD3 (CD8^dim^CD3^-^). **(C)** Strategy to analyzed gated CD8^dim^CD3^+^ cells for expression of IFN-γ or granzyme B. **(D)** Strategy to analyzed gated CD8^dim^CD3^-^ cells for expression of IFN-γ or granzyme B. **(E)** Strategy to analyzed gated CD8^hi^CD3^+^ cells for expression of IFN-γ or granzyme B. **(F)** Comparison of levels of CD8^hi^CD3^+^, CD8^dim^CD3^-^ or CD8^dim^CD3^+^ subpopulations. Shown as black circles are data for each research subject, with the mean values shown as squares.

Because there was a tendency toward reduced expression of IFN-γ and granzyme B by CD8^+^ cells of Veterans with PTSD, the phenotype of CD8^+^ cells was further examined. There were two distinct subpopulations of CD8^+^ cells: one stained brightly for CD8 and the other stained dimly. A typical scatter plot that shows these two subpopulations of CD8^+^CD4^-^ cells is in **Figure 2A**. To further characterize these cells, they were also stained for the T-cell lineage marker CD3. This revealed three subpopulations of CD8^+^ cells (**Figure 2B**): cells staining brightly for CD8 and staining positive for CD3 (CD8^hi^CD3^+^), cells staining dimly for CD8 but staining positive for CD3 (CD8^dim^CD3^+^) and cells staining dimly for CD8 but not staining for CD3 (CD8^dim^CD3^-^). For all subjects except for one control Veteran without PTSD, the CD8^hi^CD3^+^ subpopulation was the most prominent of the CD8^+^ cell subpopulations and likely constitutes the conventional CD8^+^ cells (**Figure 2F**). The CD8^dim^CD3^-^ population likely includes NK cells while the CD8^dim^CD3^+^ subpopulation is considered to be unconventional CD8^+^ cells that have been associated with chronic infection and immune fatigue (15, 16). There were no statistically significant differences in the proportion of the three CD8^+^ cell subpopulations among Veterans with PTSD or without PTSD.

**Figure 3:**
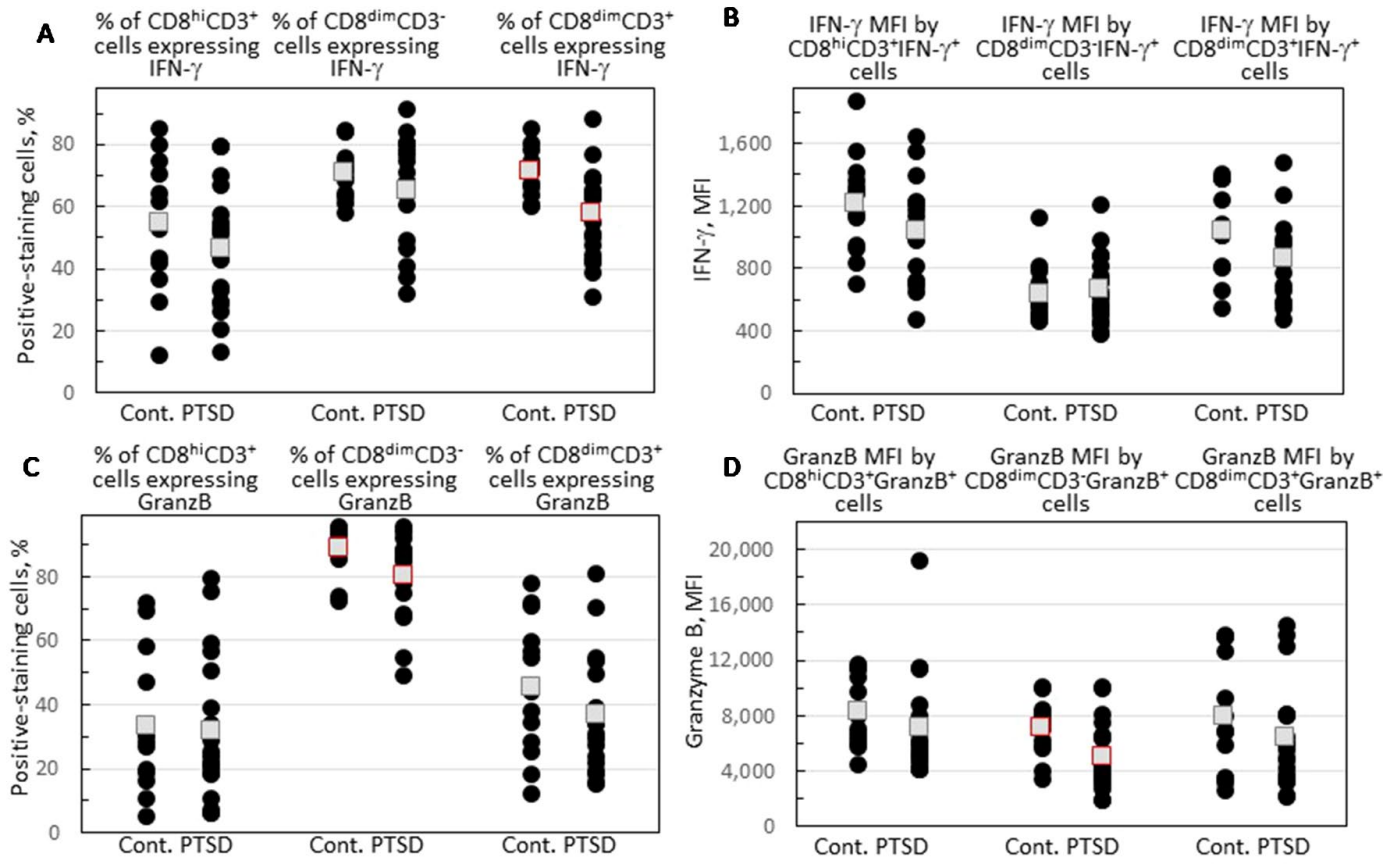
Expression of IFN-γ and granzyme B by CD8^+^ cell subpopulations. **(A)** Percentage of each of the CD8^+^CD4^-^ subpopulations that express IFN-γ. **(B)** The MFI of IFN-γ staining by each of the three gated CD8^+^CD4^-^IFN-γ^+^ cell subpopulations. **(C)** The percentage of each of the CD8^+^CD4^-^ subpopulations that express granzyme B. **(D)** The MFI of granzyme B staining by each of the three gated CD8^+^CD4^-^granzyme B^+^ cell subpopulations. Shown as black circles are data for each research subject, with the mean value shown as squares. Mean values that are statistically different between Veterans with and without PTSD are shown as red squares.

Expression of IFN-γ and granzyme B by each of the three CD8^+^ subpopulations was compared for Veterans with and without PTSD (**Figure 3**). **Figures 2C–E** show the gating strategy for this analysis. There were no differences in the proportion of either the conventional CD8^hi^CD3^+^ or the NK-containing CD3^dim^CD3^-^ cells that expressed IFN-γ between Veterans with or without PTSD (**Figure 3A**). However, the expression of IFN-γ by the less conventional CD8^dim^CD3^+^ cells was significantly lower in Veterans with PTSD (*p* = 0.002, **Figure 3A**). This reduced expression of IFN-γ by the CD8^dim^CD3^+^ cells in subjects with PTSD was not influenced by differences in the proportion of males to females among the two groups since the difference remained (*p* = 0.021) when results from only male participants were analyzed. Also, the reduced proportion of unconventional CD8^+^ T-cells expressing IFN-γ was not impacted by age as it remained statistically significant (*p* = 0.050) when only analyzing data from age-matched subjects. Sample size calculation indicated 13 subjects would be required per group for the difference levels of CD8^dim^CD3^+^ cells expressing IFN-γ to be significant, which was achieved for the control population and exceeded for the PTSD group. In addition, the effect size was large (Hedge’s *g* = 1.12), supporting the significance of the reduced level of CD8^dim^CD3^+^ cells expressing IFN-γ. The intensity of IFN-γ expression by the CD8^dim^CD3^+^ cells that stained positive for IFN-γ^+^ was also reduced and neared significance (*p* = 0.060; **Figure 3B**).

Granzyme B expression by the CD8^+^ cell subpopulations was also compared for Veterans with and without PTSD. There were no differences between Veterans with or without PTSD in the proportion of their conventional CD8^hi^CD3^+^ cells that expressed granzyme B (**Figure 3C**), although the intensity of granzyme B expression by the positive-staining cells tended to be lower for subjects with PTSD (*p* = 0.072; **Figure 3D**). There were no significant differences in the proportion of unconventional CD8^dim^CD3^+^ cells expressing granzyme B (**Figure 3C**). Most of the NK-containing subpopulation of CD8^dim^CD3^-^ cells expressed granzyme B, but the proportion of these cells that expressed the cytotoxin granzyme B was significantly lower in Veterans with PTSD compared to those without PTSD (*p* = 0.013; **Figure 3C**). Of the CD8^dim^CD3^-^ cells that expressed granzyme B, the intensity of expression was significantly reduced in Veterans with PTSD (*p* = 0.021; **Figure 3D**). The effect sizes were large for both the proportion of CD8^dim^CD3^-^ cells that expressed granzyme B (Hedge’s *g* = 0.81) and the intensity of granzyme B expression (Hedge’s *g* = 0.89). The reduced proportion of CD8^dim^CD3^-^ cells expressing granzyme B and the intensity of granzyme B expression was sufficiently prominent that it was statistically significant with a sample size well below predicted sample size calculations (25/group for the proportion of cells staining positive and 20/group for the intensity of staining). The reduced intensity of granzyme B expression in subjects with PTSD remained even when data for males only was analyzed (*p* = 0.029) or when analyzing values for age-matched subjects only (*p* = 0.048), indicating that the difference in the proportion of females in the two groups or the difference in ages of the two groups did not contribute to the results. However, significance was lost when assessing the frequency of CD8^dim^CD3^-^ cells expressing granzyme B in samples from only male subjects or only age-matched subjects, although the value for the males in the PTSD group remained lower (82.7 ± 12.2) than values for male controls (88.3 ± 8.3), and they remained lower in the age-matched analysis in the PTSD group (81.0 ± 6.9) than in the controls (90.2 ± 6.1).

Overall, these results show both statistically significant and tendencies in reductions in expression of immune mediators by CD8 subpopulations from Veterans with PTSD. The most prominent reductions were in IFN-γ expression by unconventional CD8^dim^CD3^+^ cells and in granzyme B expression by the NK-containing CD8^dim^CD3^-^ cells.

## Discussion

PTSD has been suggested to be an inflammatory disorder, although results of studies of the immune status of subjects with PTSD have varied (7, 8, 12, 13). Some of the variability could be due to differences in the demographics of the populations being studied, ranging from children to adults, or the type of trauma experienced. Also, studies often have compared blood cytokine levels between subjects with PTSD and healthy controls that have not experienced the traumatic event (7, 17, 18, 23). More rarely have studies compared populations that have experienced similar levels of trauma, with some developing PTSD and others not (8, 10). The present study was designed to be rigorously controlled in terms of the type and severity of traumatic exposure for those with or without PTSD. All enrolled subjects were Veterans of the wars in Iraq and Afghanistan, and all subjects were exposed to combat. However, some of the subjects developed PTSD and others did not. A phenotypic analysis of CD4^+^ and CD8^+^ cells was then conducted on the blood leukocytes of these subjects, including an analysis of immune mediators expressed by these cells and their subpopulations.

There was a significant reduction in the intensity of IFN-γ expression by CD4^+^ cells of Veterans with PTSD, and there was a near-significant reduction in the proportion of CD4^+^ cells expressing IFNγ. Also, there were reductions in IFN-γ and granzyme B expression by CD8^+^ cells from Veterans with PTSD that neared significance. CD8^+^ cells were more closely examined to better dissect subpopulations that may contribute to these diminished tendencies in levels of expression. This showed a significant reduction in expression of the pro-inflammatory mediator IFN-γ by the unconventional CD8^dim^CD3^+^ subpopulation in Veterans with PTSD compared to those without PTSD and a near-significant reduction in the intensity of IFN-γ expression by these cells. The other prominent result was a reduction in expression of the cytotoxic mediator granzyme B in the NK-containing CD8^dim^CD3^-^ subpopulation in the blood of Veterans with PTSD, both in the proportion of cells expressing granzyme B and in the intensity of granzyme B expression. While not statistically significant, there was a reduction in the intensity of granzyme B expression by the conventional CD8^hi^CD3^+^ T-cells that neared significance. CD8^+^ cell subpopulations have typically not been studied when comparing immune parameters in subjects with versus without PTSD, but our study indicates that differences may be revealed that would not be detectable when studying the entire CD8^+^ cell population.

It was not within the scope of this study to functionally define the CD8^+^ subpopulations. The CD8^dim^CD3^+^ cells, which had reduced IFN-γ expression in PTSD subjects, have been the least well studied. Levels of these cells have been shown to be increased as a result of latent virus infections and high pathogen burdens (14, 15). It has been suggested that these cells could be indicative of immune exhaustion. Changes in expression of immune mediators by the unconventional CD8^+^ subpopulation would be consistent with the premise that PTSD is associated with a chronic inflammatory state. Consequently, the immune status seen in PTSD subjects may indicate immune exhaustion and, thus, there is less expression of immune mediators by their T-cells. In fact, there is the dichotomy between our prior analyses of blood cytokine levels, which indicated increased levels of pro-inflammatory mediators in Veterans with PTSD (10), and the current study showing reduced expression of the pro-inflammatory mediator IFN-γ and the cytotoxin granzyme B in Veterans with PTSD. While the reason for this dichotomy is unknown, it is reasonable to speculate that cell populations other than lymphoid cells, which were analyzed in the present study, may be responsible for increased levels of circulating inflammatory mediators. At the same time, this may down-regulate the activation state of T-cell subpopulations.

There remain unresolved aspects to the present study including needing to better define the CD8^+^ subpopulations phenotypically and functionally. Although the study was tightly controlled in terms of both groups of subjects having similar levels of combat exposure, there were other variables that persisted. Veterans that met inclusion criteria were recruited without regard to gender, but those with PTSD had a greater proportion of females. This difference was not statistically significant. Nevertheless, values that were significantly reduced in the PTSD group were analyzed for males only to determine if similar conclusions would be attained without the influence of a disproportionate level of females. While the overall results remained the same and significance remained in a number of the parameters, significance was lost for some of the values suggesting the study was underpowered to analyzed immune parameters based on gender. Also, the group without PTSD was significantly older than the PTSD group. There was, however, no correlation between age and any of the immune parameters. Analysis of data from age-matched subjects resulted in the same conclusion as when analyzing all subjects, although power was lost for some of the parameters due to a reduced sample size. This suggested that age was not a factor in the immunological results, although a larger sample size would be needed to demonstrate that age did not contribute to the reduced immune activity in PTSD subjects. The tightness of the cohorts complicated recruitment of subjects into the study and, thus, the number of subjects in each group was a limitation. However, sample size calculations showed that significantly reduced immune parameters were demonstrated with sample sizes below those which would be anticipated. Furthermore, the large effect size supported the statistically significant reductions in immune parameters in PTSD subjects. It is possible that with a larger sample size, the immune parameters whose reduced levels were close to achieving significance would have become statistically significant.

Overall, this study did not see evidence of enhanced immune activation of T cells of Veterans with PTSD. Instead, the results suggested diminished activity by blood T-cell subpopulations. The reductions in expression of IFN-γ and granzyme B did not reach statistical significance when examining the CD8^+^ cell population as a whole, but did reach significance when examining the CD8^+^ subpopulations, with different mediators being reduced in different subpopulations. It should, consequently, be of interest in future studies to more closely examine CD8^+^ cell subpopulations for differences in immune activity between subjects with or without PTSD.

## Data Availability Statement

Data will be provided upon request submitted to MY at youngmr@musc.edu.

## Ethics Statement

This study was approved by the IRB of the Medical University of South Carolina, which is the IRB of record for both the Ralph H. Johnson VA Medical Center and the Medical University of South Carolina. Subjects were all explained the study, provided a written consent, and voluntarily consented to participate.

## Author Contributions

YX conducted the laboratory analyses of the blood specimens, ZW and MY were involved in the development of the study, and ZW assisted with recruitment of research subjects. MY and YX analyzed the laboratory data and all authors were involved in the preparation of the manuscript.

## Funding

This work was supported by the Clinical Sciences Research and Development Program of the Department of Veterans Affairs (I01CX000851) (MY).

## Conflict of Interest

The authors declare that the research was conducted in the absence of any commercial or financial relationships that could be construed as a potential conflict of interest.
